# Omega-3 PUFAs Suppress IL-1β-Induced Hyperactivity of Immunoproteasomes in Astrocytes

**DOI:** 10.3390/ijms22115410

**Published:** 2021-05-21

**Authors:** Emilia Zgorzynska, Barbara Dziedzic, Monika Markiewicz, Anna Walczewska

**Affiliations:** 1 Department of Cell-to-Cell Communication, Medical University of Lodz, Mazowiecka 6/8, 92-215 Lodz, Poland; barbara.dziedzic@umed.lodz.pl (B.D.); anna.walczewska@umed.lodz.pl (A.W.); 2 Department of Applied Biology, Research Institute of Horticulture, Konstytucji 3 Maja 1/3, 96-100 Skierniewice, Poland; monika.markiewicz@inhort.pl

**Keywords:** immunoproteasome, astrocytes, C/EBPs, docosahexaenoic acid, eicosapentaenoic acid, connexin 43

## Abstract

The role of immunoproteasome (iP) in astroglia, the cellular component of innate immunity, has not been clarified. The results so far indicate that neuroinflammation, a prominent hallmark of Alzheimer’s disease, strongly activates the iP subunits expression. Since omega-3 PUFAs possess anti-inflammatory and pro-resolving activity in the brain, we investigated the effect of DHA and EPA on the gene expression of constitutive (β1 and β5) and inducible (iβ1/LMP2 and iβ5/LMP7) proteasome subunits and proteasomal activity in IL-1β-stimulated astrocytes. We found that both PUFAs downregulated the expression of IL-1β-induced the iP subunits, but not the constitutive proteasome subunits. The chymotrypsin-like activity was inhibited in a dose-dependent manner by DHA, and much strongly in the lower concentration by EPA. Furthermore, we established that C/EBPα and C/EBPβ transcription factors, being the *cis*-regulatory element of the transcription complex, frequently activated by inflammatory mediators, participate in a reduction in the iP subunits’ expression. Moreover, the expression of connexin 43 the major gap junction protein in astrocytes, negatively regulated by IL-1β was markedly increased in PUFA-treated cells. These findings indicate that omega-3 PUFAs attenuate inflammation-induced hyperactivity of iPs in astrocytes and have a beneficial effect on preservation of interastrocytic communication by gap junctions.

## 1. Introduction

The immunoproteasome (iP), a key regulator of innate and adaptive immune responses [1], is a specialized, pro-inflammatory cytokine- and oxidative stress-induced form of the constitutive proteasome, significantly upregulated in immunological cells, especially in the antigen-presenting cells [2]. The β1, β2 and β5 subunits of 20S core constitutive proteasome are replaced by their inducible counterparts, i.e., iβ1/LMP2, iβ2/MECL1 and iβ5/LMP7 creating the iP, which possesses stronger than constitutive proteasome chymotrypsin-like activity in cleaving proteins, after hydrophobic amino acids [2]. Further study has revealed that the activation of iP occurs in glia and nerve cells in response to amyloid-β and in post-mortem brains of patients with Alzheimer’s disease [3]. Likewise, an abundant increase in iPs levels has been reported in the damaged retina and brain [4]. Therefore, apart from the widely known function of iP, involving the generation of peptides able to fit in the groove of MHC class I proteins, its alternative role in non-immunological tissues is still being debated.

Astrocytes, the most abundant cells in the central nervous system, are essential for the neuronal function. They support neurons by supplying vital metabolites, such as lactate and ATP, maintaining water–ion balance, protecting against oxidative stress and excitotoxicity of glutamate, and regulating synaptic processes [5,6,7]. Astrocytes are also fundamental cellular components of innate immune response to brain trauma, ischemia or infections. Under these conditions, they are transformed from a resting state into reactive phenotype that acquires cytotoxic properties to neurons and differentiated oligodendrocytes [8]. Furthermore, induced expression of numerous genes for pro-inflammatory enzymes and inflammatory mediators in these astrocytes, to a large degree induced in the NF-κB-dependent mechanism, gives rise to profuse release of pro-inflammatory cytokines that enhance progression of neuroinflammation [9].

A presence of the binding sites for the NF-κB dimer in the promoter of the iP subunit genes indicates contribution of NF-κB in the regulation of their transcription. Moreover, cumulative evidence indicates an anti-inflammatory effect of dietary omega-3 PUFAs and their derivatives in various neurological and autoimmune diseases. Recently, we demonstrated that DHA suppresses activation of NF-κB [10] and exerts an anti-oxidative effect in astrocytes [11], so in the present study we examined whether omega-3 PUFAs restrain the expression and activity of the iP. Our results confirmed that omega-3 PUFAs downregulate the gene expression of inducible catalytic subunits (iβ1 and iβ5) and activity of the iPs in reactive astrocytes. Further, we detected the participation of CCAAT/enhancer binding proteins (C/EBPs) in the regulation of iβ1 and iβ5 subunits expression by omega-3 PUFAs.

Given the extensive expression of connexins (Cxs) in astrocytes, particularly connexin 43 (Cx43) which creates hexamers embedded in the plasma membranes that form both the gap junctions and hemichannels [12], there has been a significant interest in the role that they play in the course of various neuropathologies [13]. However, the status of Cx43 throughout reactive astrogliosis is just beginning to be specified. Therefore, bearing in mind that communication between the cells and between astrocytes and extracellular compartment is crucial for brain pathologies, we extended our study and investigated whether downregulation of the iP levels and activity by omega-3 PUFAs is accompanied by changes in the Cx43 expression in activated astrocytes.

## 2. Results

### 2.1. Expression of Constitutive and Inducible Proteasome Subunits in Resting and IL-1β-Activated Astrocytes

**Figure 1:**
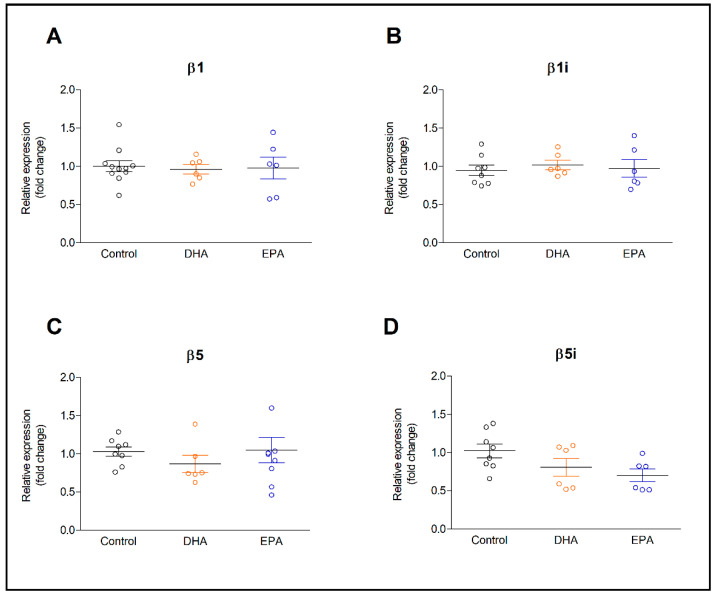
The effect of DHA and EPA (at concentration of 30 µM) on β1 (**A**), β1i (**B**), β5 (**C**), and β5i (**D**) proteasome subunits expression in rat astrocytes. Treatment protocol is described in the Methods section.

**Figure 2:**
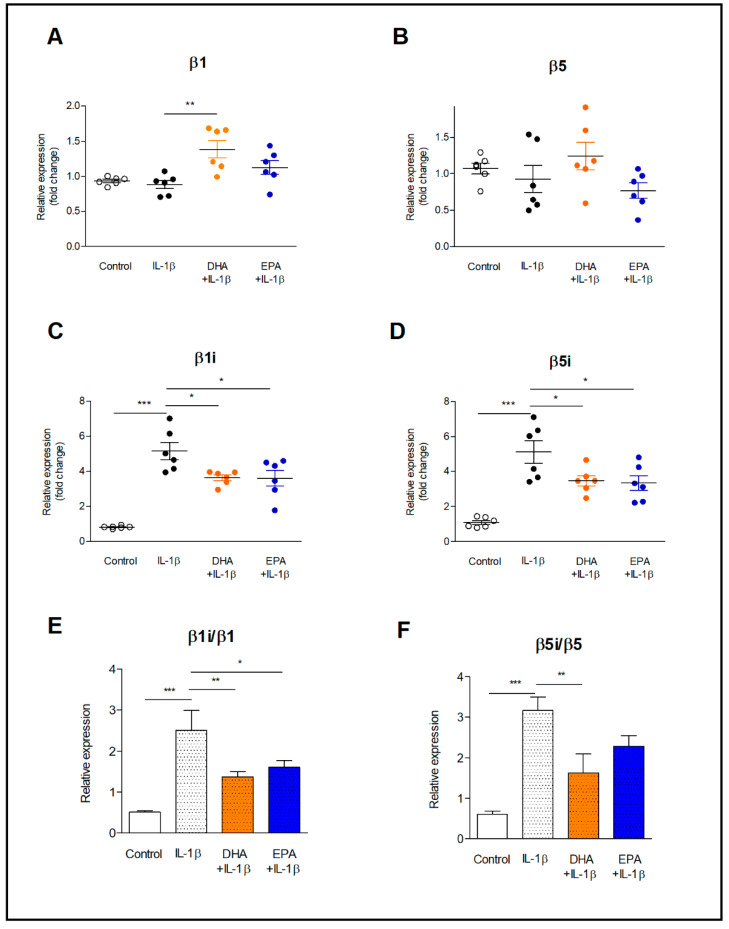
The effects of DHA and EPA on the constitutive and inducible 20S core subunit expression in astrocytes activated with IL-1β. Astrocytes were incubated with DHA and EPA (30 µM) for 24 h, followed by treatment with IL-1β (10 ng/mL) for 16 h. Total mRNA was isolated from cells by Trizol and β1 (**A**), β5 (**B**), β1i (**C**) and β5i (**D**) subunits expression was determined by quantitative real-time PCR. The ratios of inducible to constitutive proteasome subunits β1i/β1 (**E**) and β5i/β5 (**F**) expression were calculated. * *p* < 0.05, ** *p* < 0.01, *** *p* < 0.001.

The expression of β1 and β5 proteasome subunits, as well β1i and β5i immunoproteasome subunits, was determined in resting astrocytes and IL-1β-activated astrocytes after incubation with DHA and EPA for 24 h (Figure 1 and Figure 2). Modification of membrane phospholipids with both long-chain omega-3 PUFAs, had no effect on the expression of proteasomes subunits (Figure 1). However, a tendency of β5i expression to decrease after incubation with DHA and more pronounced after incubation with EPA was noticed (Figure 1D). Activation of astrocytes with IL-1β did not affect the expression of β1 and β5 constitutive subunits of 26S proteasome (Figure 2A,B). However, it contributed to a five-fold increase in the expression of β1i and β5i immunoproteasome subunits (Figure 2C,D), resulting in a significant increase in the β1i/β1 and β5i/β5 ratios (Figure 2E,F). Pre-incubation of astrocytes with DHA reduced the expression of β1i and β5i by 30% and 32% (Figure 2C,D), respectively, as well as the β1i/ β1 and β5i/β5 ratios by about a half, compared to the group treated with IL-1β without pretreatment with DHA (Figure 2E,F). Eicosapentaenoic acid, likewise DHA, lowered the mRNA levels of β1i and β5i (*p* < 0.05), although in cells treated with IL-1β without fatty acids pretreatment, the β5i/β5 ratio was 3.16, while in cells incubated with the same concentration of DHA and EPA, it was reduced to 1.63 (*p* < 0.01) and 2.29 (ns.), respectively. In contrast to the above-described results, the expression of β2 and β2i subunits in IL-1β-activated astrocytes did not change after preincubation with PUFAs (Supplementary Figure S1).

### 2.2. Proteasome Activity

**Figure 3:**
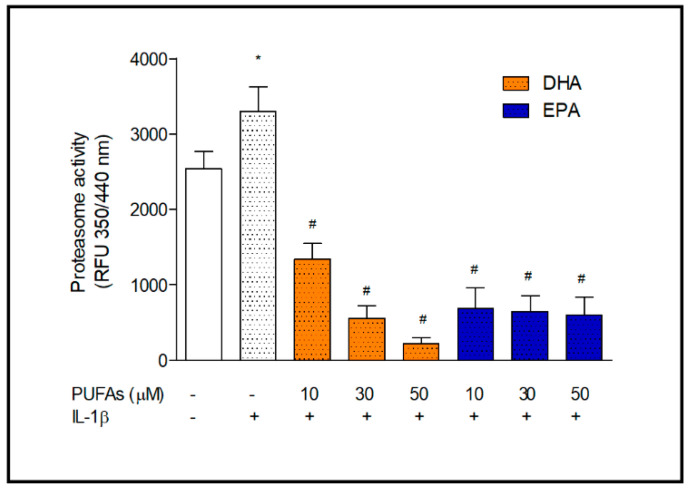
Proteasome activity in IL-1β-stimulated astrocytes incubated with various concentrations of DHA and EPA. Proteasome activity was determined in astrocyte lysates after the exposure to DHA and EPA (10, 30 and 50 µM) for 24 h followed by treatment with IL-1β (10 ng/mL) for 16 h. * *p* < 0.05 compared to unstimulated control cells; ^#^ *p* < 0.001 compared to IL-1β-treated cells.

In the next step, we determined the omega-3 PUFAs’ effect on proteasome activity that may affect the rate of protein degradation. The chymotrypsin-like activity in astrocytes treated with IL-1β was determined at various concentration of DHA and EPA in medium. Activation of astrocytes with IL-1β significantly increased the proteasome activity (*p* < 0.05). Pre-incubation of the cells with increasing concentrations of omega-3 PUFAs inhibited chymotrypsin-like activity; however, the course of the inhibition for these fatty acids was different. Docosahexaenoic acid inhibited proteasome activity in a dose-dependent manner and, at the lowest concentration, decreased proteasome activity by 60%. While EPA at the lowest concentration more strongly reduced the activity (almost by 80%), whereas higher concentrations of EPA did not make any further changes in the proteasome activity (Figure 3).

### 2.3. C/EBPs Transcription Factors Activation

**Figure 4:**
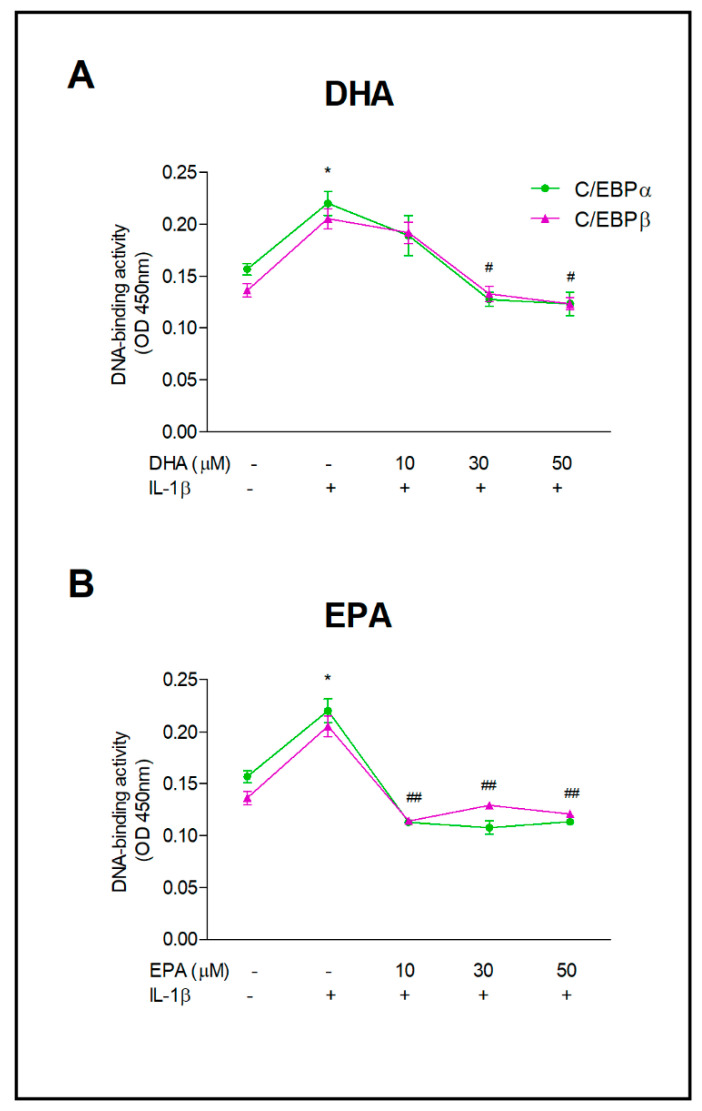
DNA binding activity of C/EBPα and C/EBPβ transcription factors in astrocytes incubated with increasing doses of DHA (**A**) and EPA (**B**). After 24 h incubation with omega-3 PUFAs, cells were treated with 10 µg/mL IL-1β for 16 h. In nuclear fraction of these astrocytes, C/EBPα and C/EBPβ activity was determined colorimetrically. Indicators for statistical significance are common for both transcription factors. * *p* < 0.001 compared to unstimulated control cells; ^#^ *p* < 0.01, ^##^ *p* < 0.001 compared to IL-1β- treated cells.

Since the genes for β1i (PSMB9) and β5i (PSMB8) possess the CAAT-box in their proximal promoters, we examined whether PUFAs’ reduction in these inducible subunits’ expression is associated with changes in the activity of C/EBPα and/or C/EBPβ transcription factors, responsible for binding to transcriptional enhancers. As expected, exposure of astrocytes to IL-1β significantly increased binding of the C/EBPs to DNA (0.205 ± 0.009; *p* < 0.001) (Figure 4). Pre-incubation of astrocytes with PUFAs decreased the activity of both transcription factors similarly, although the course of binding inhibition to DNA by DHA and EPA differed. Pre-incubation of cells with DHA at 10, 30 and 50 μM reduced C/EBPβ activity gradually to the levels of 0.192 ± 0.020, 0.133 ± 0.007 and 0.123 ± 0.005, respectively (Figure 4A). The effect of DHA incubation on C/EBPα activity was almost identical. Pre-incubation with EPA strongly inhibited both α and β C/EBP transcription factors binding to DNA just from the lowest concentration (*p* < 0.001) and higher concentrations of EPA did not alter their binding to DNA (Figure 4B).

### 2.4. Expression of Connexin 43

**Figure 5:**
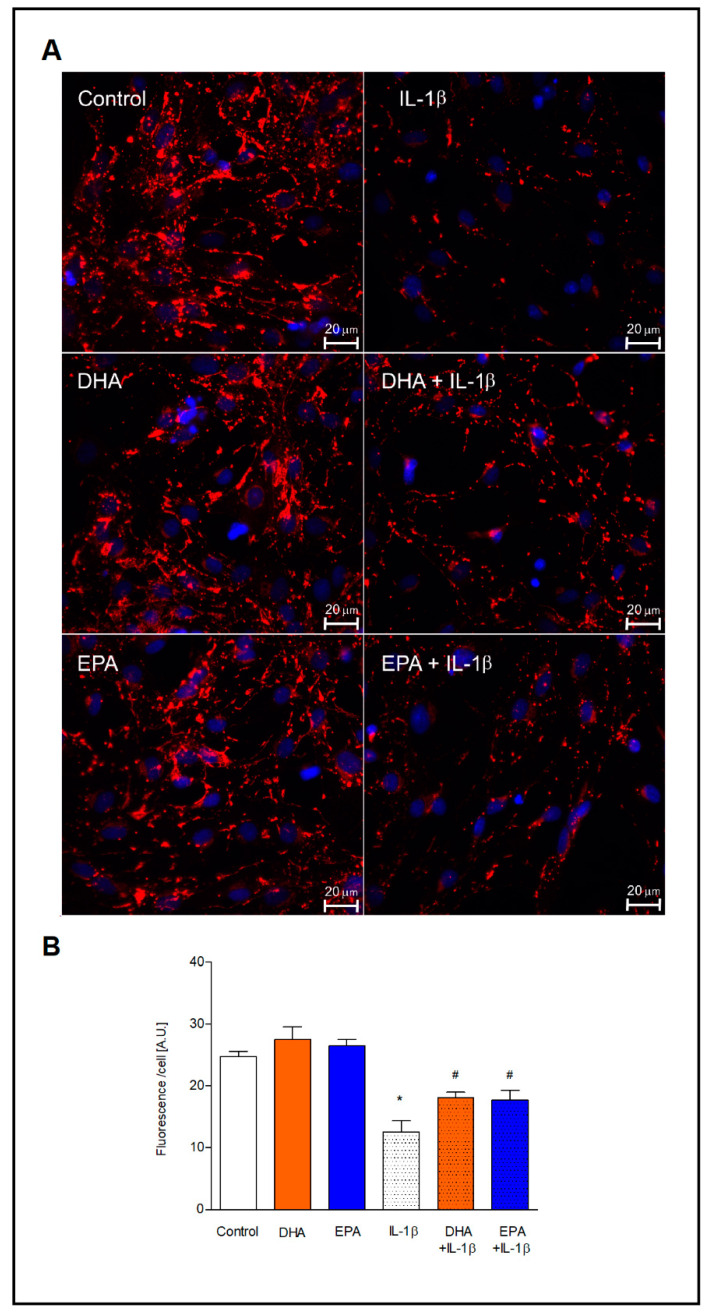
Expression of connexin 43 in astrocytes. (**A**) Representative fluorescence images depicting the double immunostaining of connexin 43 (red) with DAPI (blue). The cells were incubated with 30 µM of DHA and EPA for 24 h and then treated with 10 ng/mL 1L-1β for 16 h. (**B**) Quantification of the immunofluorescence signal in the cells. The number of cells in the field was counted and an average fluorescence intensity value per cell was calculated * *p* < 0.001 compared to unstimulated control cells, ^#^ *p*< 0.05 compared to IL-1β-treated cells.

The impact of EPA and DHA on the expression of Cx43, the most abundant hexamer in astrocyte cell membrane was verified by immunofluorescence staining (Figure 5). The expression of Cx43 in resting astrocytes was not affected after incubation with both omega-3 PUFAs. Activation of astrocytes with IL-1β decreased markedly the Cx43 levels relative to unstimulated cells (*p* < 0.001), while pre-incubation with both PUFAs resulted in preserving high levels of this protein after IL-1β treatment. After pre-incubation with EPA and DHA, the increase in the fluorescence intensity of Cx43 was almost identical (by 29% and 31%, respectively) compared to the cells treated with IL-1β without pretreatment with omega-3 PUFAs.

## 3. Discussion

A large amount of evidence, from cellular and in vivo studies has suggested anti-inflammatory effects of omega-3 PUFAs [14,15]. As a result of cell-culture supplementation with DHA and EPA or following their ingestion, plasma membrane enrichment readily occurs [11,16]. Long-chain omega-3 PUFAs incorporated into membranes, primarily at the sn-2 position of phospholipids, change physicochemical properties of membranes and an operation of membrane proteins [17]. Consequently, omega-3 PUFAs mediate the effects at multiple stages of cellular complexity and organization, affecting the signaling pathways. They lead to activation or inhibition of transcription factors, thereby ultimately impacting gene expression. Apart from the above-mentioned effects of omega-3 PUFAs in cells, the increased proportion of respective omega-3 fatty acids (DHA and EPA) to omega-6 arachidonic acid, being normally in excess in the membranes, results in a lower production of pro-inflammatory eicosanoids in favor of DHA- and EPA-oxygenated derivatives, which inhibit neutrophil recruitment, enhance their clearance from the nervous tissue, and help to restore tissue homeostasis [18].

In the iP, three proteolytically active subunits of 20S core rings of the constitutive proteasome are replaced on their homological cytokine-induced subunits, iβ1/LMP2, iβ2/MECL1 and iβ5/LMP7 [19,20]. A main role of iPs is hydrolysis of pathogen peptides available for MHC class I antigen presentation in immune competent cells [21], consequently, their absence is associated with impaired acting of CD8+ T-cytotoxic cells, especially in viral infections [22]. However, the paradigm of the iP function, solely as a participant in CD8+ T-cell mediated resolution of intracellular infection, is outdated due to an increased number of studies which confirm a constitutive and induced expression of iPs in non-immunological organs, such as the heart [23], kidneys [24], lung and liver [25]. The iPs are formed, as well, in microglia in response to inflammatory signals, and in astrocytes during sustained pro-inflammatory cytokine release by activated microglia, primarily IFNs and TNFα [26]. Indeed, the inhibition of iP activity was associated with decreased IFNγ-dependent expression of pro-inflammatory cytokines in microglia [27] and attenuation of disease progression in murine models of Alzheimer’s disease [3] and experimental encephalomyelitis [28]. Accordingly, the upregulation of the iP expression over the course of Alzheimer and Huntington diseases [29,30] and in neurons of aging hippocampus [31] were reported. Nevertheless, whether the non-immunological function of iP in astrocytes, among others, in removing ROS and oxidated and poly-ubiquitinated proteins is still discussed [32,33]; notably, astrocytes do not readily present antigens in vivo [34,35]. However, Rostami et al. [36] have recently reported that α-synuclein accumulated in astrocytes triggered expression of specific co-stimulatory molecules for activation of CD4+ T-cells in the course of antigen presentation.

Here, we demonstrate that the expression of inducible iβ1/LMP2 and iβ5/LMP7 subunits in IL-1β-activated astrocytes, not enriched with omega-3 fatty acids, was highly upregulated and the activity of the iP was much higher compared to non-stimulated astrocytes. Conversely, in astrocytes incubated with omega-3 PUFAs prior IL-1β stimulation, the subunits’ expression decreased. Furthermore, the proteosome activity dropped in a dose-dependent manner after incubation of cells with DHA, and it was inhibited to the same extent by EPA in a range of concentrations. A binding of IL-1β to TLR/IL-1 type of receptor (IL-1R) elicits signaling that cascades through the MyD88/IRAKs complex, which activates the IκB kinase (IKK)-NF-κB complex and MAPK kinases [37]. These pathways promote NF-κB and effector MAPK transcription factors, making them available for the nuclear DNA. Genes for iβ1/LMP2 and iβ5/LMP7 subunits are located in the MHC class II region of chromosome 6 (6p21.3), and both are regulated by similar transcription factors, among others, by NF-κB and AP-1 [38,39]. We have recently reported that DHA inhibits the activity of NF-κB and AP-1 transcription factors in astrocytes stimulated with IL-1β [10]. In this work, both DHA and EPA downregulated the expression of inducible iβ1/LMP2 and iβ5/LMP7 subunits at a similar intensity, so we assume that EPA as DHA also inhibits these transcription factors.

Next, we tested whether transcription factors C/EBPα and C/EBPβ from the family of CAAT-enhancer-binding proteins can be involved in the regulation of inducible subunits’ expression. C/EBPα and C/EBPβ, with four other structurally and functionally homologous transcription factors, belong to the family of transcription factors containing a C-terminal basic region-leucine zipper domain [40]. C/EBPs bind to the CAAT motif in DNA and through the transactivation domain, with transcription coactivators, such as ATP-dependent chromatin-remodeling enzymes [41]. Importantly, β1 and β5 genes carry the CAAT-box in their promoters [42]. The expression and activation of C/EBPs are regulated by a variety of extracellular signals, frequently by pro-inflammatory signals, such as IL-1, IL-6 and LPS [43,44] in a complex manner, by post-translational modifications and protein–protein interactions [45,46,47]. It has been shown that the phosphorylation of C/EBPs at different sites by number of protein kinases, including PKC [48], MAPKs [49] and calcium-calmodulin-dependent protein kinase (CaMKII) [50], affects functions of C/EBPs from an attenuation of their binding to DNA [48] to an increase in their transcriptional activity [50]. Based on the current binding assay results, we cannot propose the state of C/EBPs phosphorylation which leads to a reduction in their binding to DNA. However, ex vivo and in vitro experiments have shown that C/EBPβ acquires DNA-binding function when it is phosphorylated at Thr188 first by MAPK, and then at Ser184 and Thr179 by glycogen synthase kinase 3β (GSK3β) [51]. Since Akt kinase phosphorylates GSK3 in a highly conserved N-terminal regulatory site inactivating GSK3 [52] and it is activated by DHA [16], reduced binding of C/EBP to DNA by omega-3 PUFA in IL-1β-stimulated astrocytes may be caused by inhibition of GSK-3 activity [53]. Considering our earlier and present results, we conclude that enrichment of astrocytic membranes with omega-3 PUFAs downregulates the expression of iβ2 and iβ5 subunits by reducing C/EBPα and C/EBPβ activity, as well NF-κB inhibition [10].

A key factor in the astroglial network function is an efficient operation of embedded in plasma membranes hexamers of connexins. When they are associated head-to-head, they form gap junctions (GJs), thus creating the astrocytic syncytium [54] or exist in the cell membrane as hemichannels (HCs), allowing low-weight molecules exchange between the astrocyte cytoplasm and extracellular compartment [13]. Since Cx43 is most abundant in astrocytes [55] and form both gap junctions and hemichannels, we investigated whether a downregulation of iPs by omega-3 PUFAs may prevent a degradation of Cx43 in activated astrocytes. The result confirmed that enrichment of plasma membranes with omega-3 PUFAs prevent a dramatic loss of Cx43 induced by IL-1β. Similarly, the inhibition of IL-1β-mediated decrease in Cx43 content by DHA was demonstrated earlier in cardiomyocytes [56]. Notwithstanding, PUFAs per se, without pro-inflammatory insult, did not significantly increased the Cx43 levels, which is in opposition to the result reported by Champeil-Potocar et al. [57]. The authors have shown enhanced GJ coupling, determined by scrape-loading-Lucifer yellow dye transfer and higher levels of phosphorylated Cx43 in DHA-enriched astrocytes. These discrepancies in results may arise from the other protocol of astrocyte treatment with PUFA. In that study, astrocytes were incubated with DHA together with antioxidants for 10 days, not for 24 h as in our study, which may reflect entirely different mechanism of PUFA action and different places of Cx43 phosphorylation.

A connexin export to the plasma membrane, assembly, gating and degradation in the cell are complex and not fully understood; however, they all appear to be regulated via post-translational modification, primarily by phosphorylation of GJ domains. The sequence of phosphorylation at specific sites of Cx43 molecule is performed by numerous kinases, such as Akt, PKA and CK1 which stabilize the scaffold of GJ, or by PKC, MAPKs, and Src, which causes GJs disaggregation followed by their degradation by various mechanisms [58,59]. Since PKC can be inhibited by both omega-3 PUFAs in neurons [60,61], consequently, they can inhibit of Cx43 degradation by PKC-dependent mechanism in astrocytes [62,63]. It has been demonstrated that the major mechanism of Cx43 degradation is ubiquitin-mediated proteosome proteolysis [64], particularly induced by the pro-inflammatory signal [65]; therefore, we suggest that, next to anti-inflammatory effects during inflammatory cytokine-impact, omega-3 PUFAs also prevent the connexon scaffold from disaggregation and proteasomal degradation.

In conclusion, our results indicate that omega-3 PUFAs inhibit the expression of iβ1/LMP2 and iβ5/LMP7 immunoproteasome subunits through a reduction in C/EBPα and C/EBPβ binding to their gene promotors. Furthermore, enhanced by IL-1β iP activity was markedly reduced in DHA and EPA-enriched astrocytes. Taken together, these findings suggest that long-chain omega-3 PUFAs may have a beneficial effect due to their prevention of excessive proteasomal protein degradation, such as Cx43; this is essential for proper functioning of the astroglia network and cellular homeostasis.

## 4. Materials and Methods

### 4.1. Culture of Primary Astrocytes and Treatment

Astrocytes were isolated from the cortex of 1–2-day-old rat pups using the modified method described previously [10]. The protocol was approved by the Local Ethics Committee. Briefly, after decapitation, the brain was dissected, washed with PBS (Sigma-Aldrich, St. Louis, MO, USA) and the meninges were stripped off. The cortices were dissociated by trituration in sterile tubes with DMEM (Gentaur, Sopot, Poland). The suspension was filtered forcefully through 180 µm and 30 µm Nitex mesh (Merck Millipore, Darmstadt, Germany), and centrifuged at RT. The cell pellet was suspended in DMEM:F12 medium containing 10% FBS (Sigma-Aldrich, St. Louis, MO, USA) and supplemented with antibiotics (Gentaur, Sopot, Poland) (100 units/mL penicillin, 100 μg/mL streptomycin). The cells were counted, seeded in 75 cm^2^ tissue culture flasks at density 2 × 10^5^ cells/cm^2^, and cultured in humidified atmosphere of 95% air and 5% CO_2_ at 37 °C. The medium was changed every two days up to a confluence of 70–80%; then the flasks were shaken to detach non-astrocytic cells. Residual cells were digested with 0.25% trypsin, counted, plated into 75 cm^2^ flasks and cultured until they were 80% confluent. The purity of the cortical astrocytes was greater than 98%, as determined by immunofluorescence assay with anti-GFAP (ThermoFischer Scientific, Waltham, MA, USA). Before each experiment, astrocytes were seeded on culture dishes in DMEM containing 10% FBS and supplemented with antibiotics (100 units/mL penicillin, 100 μg/mL streptomycin) for 48 h. Then, the medium was changed to DMEM w/o FBS containing 30 μM DHA (Sigma-Aldrich, St. Louis, MO, USA) or EPA (Sigma-Aldrich, St. Louis, MO, USA) for 24 h followed by treatment with 10 ng/mL IL-1β (Sigma-Aldrich, St. Louis, MO, USA) for 16 h. To determine a dose-dependent effect of omega-3 PUFAs on C/EBPs and proteasome activity, the cells were pre-incubated for 24 h with 10, 30 and 50 μM DHA and EPA. Control cells were cultured in DMEM w/o FBS.

### 4.2. RNA Isolation and Gene Expression Analysis

To examine the expression of proteasome and immunoproteasome subunits in astrocytes, RNA was isolated using the Trizol RNA reagent (Invitrogen, Thermo Fischer Scientific, Waltham, MA, USA), then purified with the PureLink RNA Mini Kit (Invitrogen, Thermo Fischer Scientific, Waltham, MA, USA) according to the manufacturer’s instructions. Concentration and purity of the total RNA were examined using an Epoch spectrophotometer (BioTek, Highland Park, VT, USA) in duplicate. From each sample, 1 µg of RNA was reverse-transcribed using M-MLV reverse transcriptase (Promega, Madison, WI, USA) and oligo(dT)_15_ primer (Promega, Madison, WI, USA) in a 25 µL reaction volume. Obtained cDNA samples were used to the gene expression analysis performed using the quantitative real-time PCR (qRT-PCR) technique for the genes encoding the *β1*, *β1i* (*LMP2*), *β2*, *β2i* (*MECL1*), *β5* and *β5i* (*LMP7*). Sequences of the primers are presented in Table 1 [31,66,67]. The constitutively expressed *GAPDH* gene was applied as a reference gene. Quantitative RT-PCR was carried out in Rotorgene 6000 machine (Corbett Research, Bath, United Kingdom) using KAPA^TM^ SybrFast qPCR Master Mix (Kapa Biosystems, Amsterdam, The Netherlands), according to manufacturer’s instructions, in a total volume of 20 µL and 1/10 cDNA dilution for each tested sample. The annealing temperature for all primers was 58 °C. The melting curves of the amplified products were analyzed at the end of each PCR, the analysis being carried out at 72–95 °C, with temperature raised by 1 °C/5 s. Four ten-fold dilutions of cDNA were run together with analyzed samples for a calculation of the standard curve (correlation coefficient > 0.99) and the PCR efficiency. The relative quantification of mRNA level of tested genes was read out from the standard curve and normalized to the GAPDH gene. All calculations were done using Rotor-Gene 6000 Series Software 1.7 (Corbett Research, Bath, United Kingdom). Results were obtained from two independent experiments each in triplicate.

### 4.3. Proteasome Activity Assay

Chymotrypsin-like activity of proteasome in astrocytes was quantified using Abcam kit (ab107921, Abcam, Cambridge, United Kingdom) utilizing a fluorescent AMC (7-amino-4-methylcoumarin)-tagged peptide substrate (Succ-LLVY-AMC). In brief, cells after treatment were lysed in 0.5% NP-40, centrifuged at 15,000× *g* rpm in 4 °C and assayed with and without proteasome inhibitor MG132. Fluorescence was measured at 350/440 nm (excitation/emission) in the microplate reader (Perkin-Elmer, Waltham, MA, USA). The data were obtained from two independent experiments carried out in triplicate.

### 4.4. Determination of C/EBPs Activation

Nuclear proteins were extracted from astrocytes using NE-PER Nuclear and Cytoplasmic Extraction Reagents (Thermo Fischer Scientific, Waltham, MA, USA) according to the manufacturer’s instruction. In order to make quantitative measurements of C/EBPα and C/EBPβ transcription factors activation, a colorimetric Abcam kit (ab207199, Abcam, Cambridge, United Kingdom) was used. Briefly, nuclear extracts were added to a 96-well plate pre-coated with a specific double stranded DNA sequence containing the consensus binding sites for C/EBP α/β. Primary antibodies that recognize the epitopes of C/EBP α/β bound only to the target sequences of DNA were added into wells and incubated at RT for 1 h. After washing and 1-h incubation with HRP-conjugated secondary antibodies, developing solution was added and the reaction was finished by adding Stop Solution. The absorbance at OD 450 nm was read within 5 min. Three independent experiments performed in triplicate were carried out.

### 4.5. Immunofluorescence Microscopy

Astrocytes seeded on glass coverslips were cultured as described earlier. After washing with PBS, the cells were fixed with 4% paraformaldehyde (Avantar Performance Materials, Gliwice, Poland) in PBS at RT and permeabilized with 0.2% Triton X-100 (Avantar Performance Materials, Gliwice, Poland). Next, they were blocked with 2% serum in PBS and incubated overnight at 4 °C with anti-connexin 43 antibodies (1:400) (Thermo Fischer Scientific, Waltham, MA, USA). Next day, Alexa 594-conjugated secondary antibodies (Thermo Fisher Scientific, Waltham, MA, USA) were applied at RT, in the dark, for two hours. Finally, the cells were co-stained with the DNA-binding dye 4,6-diamidino-2-phenylindole (DAPI) (Thermo Fischer Scientific, Waltham, MA, USA), and the coverslips were mounted on glass microscope slides using ProLong Gold antifade reagent (Thermo Fischer Scientific, Waltham, MA, USA). Fluorescent images were acquired with an AxioExaminer epifluorescence microscope (Carl Zeiss, Oberkochen, Germany) equipped with a water immersion objective. Images were captured at 40× magnification.

### 4.6. Statistical Analysis

The results are means ± SEM. All data were analyzed using the GraphPad Prism 6.0 software (San Diego, CA, USA). Statistical significance was determined by one-way ANOVA with Bonferroni correction. For nonparametric data, the Kruskal–Wallis test, followed by the Dunn’s multiple comparison test, was applied. The level of significance was set at *p* < 0.05.

## Figures and Tables

**Table 1:** Sequences of the primer pairs used for the real time PCR experiments.

Subunit	Sequence
**β1**	Forward 5′-CTTATGCCTTCAACGGAGGT-3′
	Reverse 5′-GTGTCTGAAGCAACGATGGA-3′
**β1i**	Forward 5′-GACGGGAGAAGTCCACACC-3′
	Reverse 5′-ATCAGAGCCCACCACGAC-3′
**β2**	Forward 5′-GAGGGCAGTGGAGCTTCTTA-3′
	Reverse 5′-AGGTGGGCAGATTCAAGATG-3′
**β2i**	Forward 5′-TTCCAGCCAAACATGACG-3′
	Reverse 5′-AGTGATCACACAGGCATCCA-3′
**β5**	Forward 5′-CTCCAAACTGCTTGCCAAC-3′
	Reverse 5′-CCTGTTCCCCTCACTGTCTA-3′
**β5i**	Forward 5′-CGCAGGAAGTTACATTGCTAC-3′
	Reverse 5′-CCATTCCGCAGATAGTATAGCC-3′
**GAPDH**	Forward 5′-TGACAACTTTGGCATCGTGG-3′
	Reverse 5′-TACTCCTTGGAGGCCATGT-3′

## Data Availability

All data are included in this article.

## Supplementary Materials

The following are available online at https://www.mdpi.com/article/10.3390/ijms22115410/s1.
