# Supplementary figures and images for: Omega-3 PUFAs Suppress IL-1β-Induced Hyperactivity of Immunoproteasomes in Astrocytes
Source: Int J Mol Sci. 2021 May 21;22(11):5410. doi: 10.3390/ijms22115410 (PMC8196670; doi:10.3390/ijms22115410)

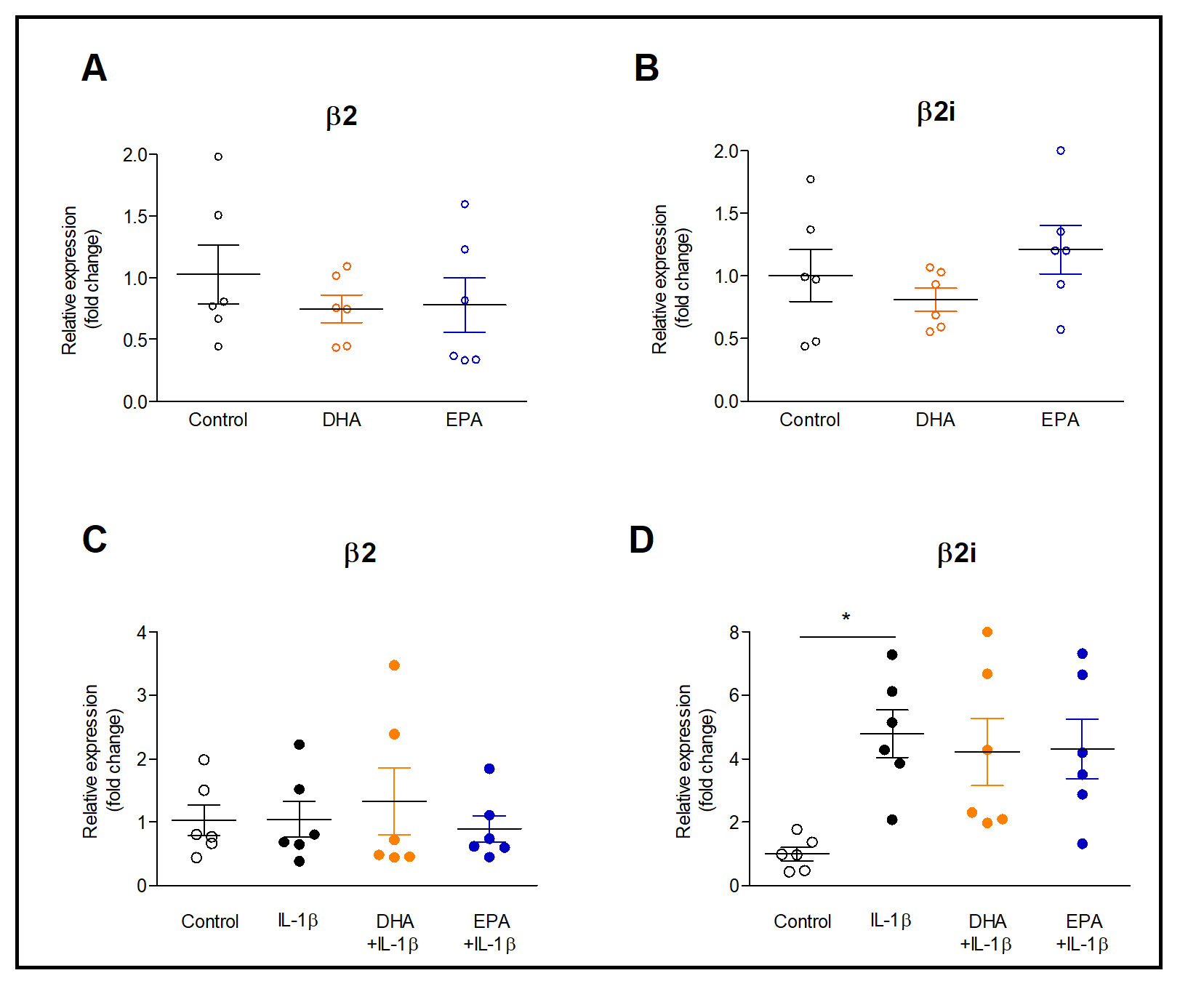

Supplement: Supplementary file 1 [file ijms-22-05410-s001.zip › ijms-1223037-supplementary.tif]
